# Differing Behavioural Responses of *Bemisia tabaci* MEAM1 and MED to Cabbage Damaged by Conspecifics and Heterospecifics

**DOI:** 10.1038/srep35095

**Published:** 2016-10-12

**Authors:** Hailong Kong, Yang Zeng, Wen Xie, Shaoli Wang, Qingjun Wu, Xiaoguo Jiao, Baoyun Xu, Youjun Zhang

**Affiliations:** 1 Department of Plant Protection, Institute of Vegetables and Flowers, Chinese Academy of Agricultural Science, Beijing 100081, China; 2 School of Horticulture and Plant Protection, Yangzhou University, Yangzhou, Jiangsu 225009, China; 3 College of Life Science, Hubei University, Wuhan 430062, China

## Abstract

The whitefly *Bemisia tabaci* is a serious pest with an extensive host range. Previous research has shown that *B. tabaci* is a species complex with many cryptic species or biotypes and that the two most important species are MEAM1 (Middle East-Minor Asia 1) and MED (Mediterranean genetic group). MEAM1 and MED are known to differ in their preference for cabbage, *Brassica oleracea*, as a host plant, however, the mechanism underlying this preference is unknown. In the current study, a host choice experiment showed that MED prefers to settle and oviposit on undamaged cabbage plants rather than MED-damaged cabbage plants. However, MEAM1 prefers MED-damaged cabbage plants to undamaged plants and does not exhibit a significant preference for undamaged or MEAM1-damaged cabbage plants. On the basis of gas chromatography-mass spectrometry (GC-MS) analysis, the following volatiles were released in larger quantities from Q-damaged cabbage plants than from undamaged plants: 2-ethyl-1-hexanol, benzenemethanol, (E)-2-decenol, benzaldehyde, nonanal, acetic acid geraniol ester, 4-hydroxy-4-methyl-2-pentanone, decane, and α-longipinene. Only one volatile, 4-hydroxy-4-methyl-2-pentanone, was released in greater quantities from MEAM1-damaged cabbage plants than from undamaged plants. Our results suggest that differences in herbivore-induced host volatile release may help explain the differences between the preference of *B. tabaci* MEAM1 and MED for cabbage as a host.

Herbivore-induced plant volatiles (HIPVs) are volatile semiochemicals that plants emit either at the site of herbivore damage or at a distance from this site^1^. Plants often increase their synthesis and emission of HIPVs when damaged by herbivores^2^. Although HIPVs can benefit the plant by attracting natural enemies of the herbivores and by repelling other herbivores, HIPVs can also harm the plant by attracting conspecific or heterospecific herbivores^3^. HIPVs are an important aspect of insect-plant interactions, and understanding their effects is important not only from an ecological and evolutionary perspective but also for the development of novel crop-protection strategies. Some volatiles, for example, have been used to mass trap pests or to repel pests from food and oviposition sites^4^,^5^. The use of HIPVs for integrated pest control requires an understanding of their effects on both natural enemies and on conspecific and heterospecific herbivores.

The whitefly *Bemisia tabaci* (Hemiptera: Aleyrodidae), a highly destructive agricultural pest, is a species complex composed of many morphologically indistinguishable biotypes. The two most invasive and destructive species are MEAM1 (Middle East-Minor Asia 1) and MED (Mediterranean genetic group)^6^,^7^,^8^. *B. tabaci* is widely distributed in the tropics and subtropics and has become a worldwide pest^9^. In China, *B. tabaci* was first recorded in the late 1940 s^10^, but the damage it inflicted on crops was not considered serious until the introduction of MEAM1 in the 1990 s^11^. In recent years, MED has invaded China and has now displaced MEAM1 in many areas^12^,^13^. Because *B. tabaci* MEAM1 and MED are resistant to insecticides and are not effectively controlled by other traditional methods, alternative methods of control are being investigated^14^.

*B. tabaci* can infest more than 600 species of plants, including cabbage, tomato, cotton, cucumber, sweet pepper, and other economically important species^15^. *B. tabaci* larvae have very limited mobility, and they therefore rely on maternal choice to determine their host plant^14^. It follows that modification of host plant choice, i.e., of maternal behaviour, could be useful for controlling this pest. Jiao *et al.*^14^,^16^ found that the proportion of whiteflies that settled and oviposited on cabbage was significantly higher for MEAM1 than for MED. Thus, the attraction to cabbage was greater for MEAM1 than MED. However, little is known about the mechanism underlying the differences in the selection of cabbage by MEAM1 and MED. We hypothesise that HIPVs may help explain why MEAM1 and MED differ in this preference. To test this hypothesis, we compared both the preference of MEAM1 and MED whiteflies for undamaged and damaged cabbage plants and the volatile compounds released by these plants. In this study, the term “damaged plants” refers to plants damaged by conspecifics (or heterospecifics), i.e., by the same (or another) species that was tested for preference.

## Results

### The preferences of *B. tabaci* B and Q adults for settling and ovipositing on undamaged cabbage plants vs. cabbage plants damaged by conspecifics and heterospecifics

**Figure 1:**
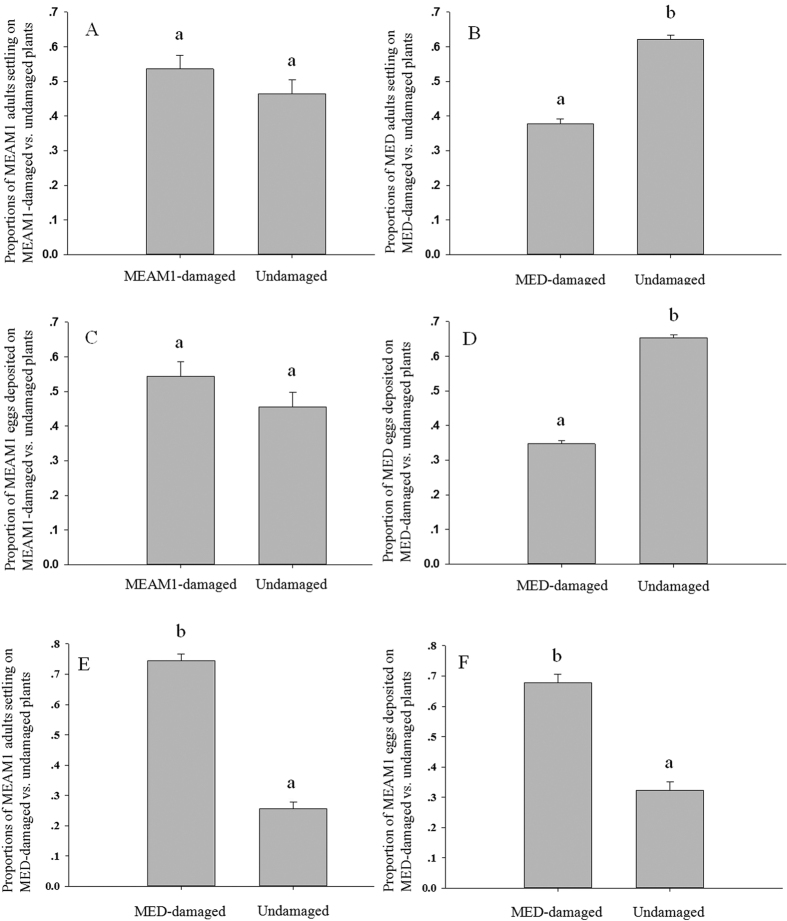
The preferences of *B. tabaci* MEAM1 and MED adults for undamaged cabbage plants vs. cabbage plants damaged by conspecifics in two-choice bioassays. (**A**) The proportions of MEAM1 whiteflies that settled on MEAM1-damaged vs. undamaged cabbage plants. (**B**) The proportions of MED whiteflies that settled on MED-damaged vs. undamaged cabbage plants. (**C**) The proportions of eggs deposited by MEAM1 whiteflies on MEAM1-damaged vs. undamaged cabbage plants. (**D**) The proportions of eggs deposited by MED whiteflies on MED-damaged vs. undamaged cabbage plants. Values are means ± SE. (**E**) The proportions of MEAM1 whiteflies that settled on MED-damaged vs. undamaged cabbage plants. (**F**) The proportions of eggs deposited by MEAM1 whiteflies on MED-damaged vs. undamaged cabbage plants. Values are means ± SE.

The proportion of B whiteflies that settled on cabbage plants did not significantly differ between MEAM1-damaged and undamaged plants (Fig. 1A) (t = 1.51, df = 16, *P* > 0.05); there was a tendency, however, for more MEAM1 whiteflies to settle on MEAM1-damaged plants than on undamaged plants. In contrast, a significantly smaller proportion of MED whiteflies settled on MED-damaged than on undamaged cabbage plants (Fig. 1B) (t = −15.77, df = 16, *P* < 0.001). For. MEAM1 whiteflies, the proportion of eggs deposited did not significantly differ between MEAM1-damaged and undamaged cabbage plants (Fig. 1C) (t = 1.27, df = 16, *P* > 0.05), but there was a tendency for larger numbers on the damaged plants. MED whiteflies, in contrast, deposited significantly fewer eggs on MED-damaged than on undamaged cabbage plants (Fig. 1D) (t = −13.18, df = 16, *P* < 0.001).

In addition, a significantly higher proportion of MEAM1 whiteflies settled on MED-damaged than on undamaged cabbage plants (Fig. 1E) (t = 8.57, df = 16, *P* < 0.001), and MEAM1 whiteflies deposited significantly more eggs on MED-damaged than on undamaged cabbage plants (Fig. 1F) (t = − 15.64, df = 16, *P* < 0.001).

### Volatiles released by undamaged, MEAM1-damaged, and MED-damaged cabbage plants

**Figure 2:**
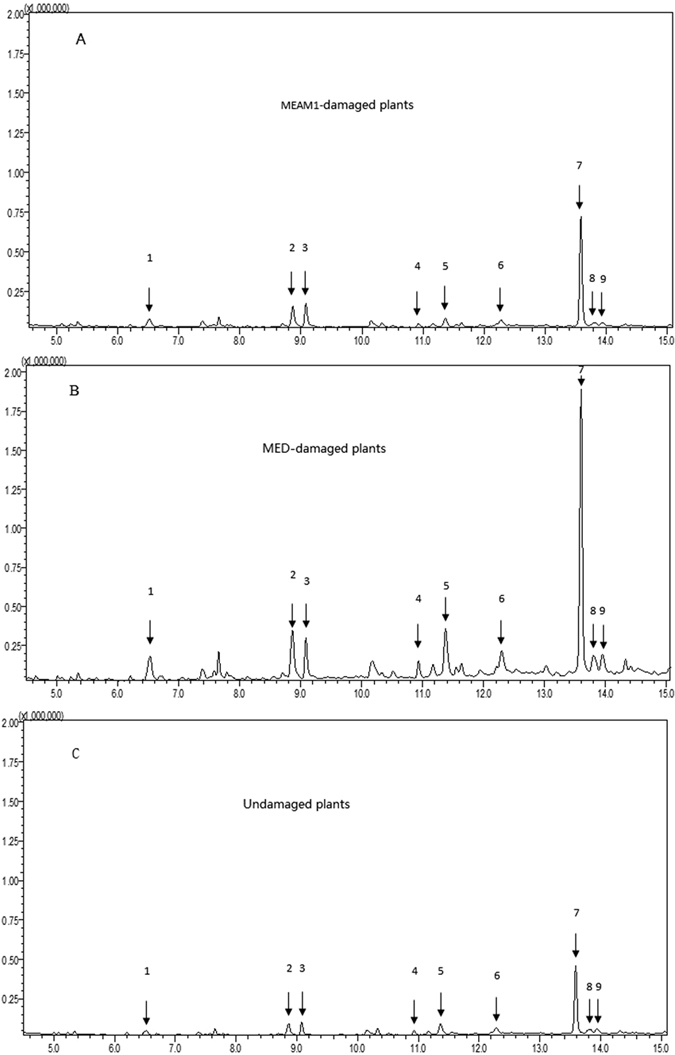
Total ion chromatograms of volatile compounds released by undamaged cabbage plants and cabbage plants damaged by the MEAM1 or MED species of *B. tabaci*. Those compounds that differed significantly in quantity among the three types of cabbage plants were: 1 = (E)-2-decenol; 2 = 4-hydroxy-4-methyl-2-pentanone; 3 = benzaldehyde; 4 = decane; 5 = acetic acid geraniol ester; 6 = 2-ethyl-1-hexanol; 7 = α-longipinene; 8 = benzenemethanol; and 9 = nonanal.

According to GC-MS chromatograms, the volatiles released by MED-damaged and undamaged cabbage plants differed quantitatively (Fig. 2; Table 1). MED-damaged cabbage plants emitted significantly more of the following volatiles than undamaged plants: 2-ethyl-1-hexanol, benzenemethanol, (E)-2-decenol, benzaldehyde, nonanal, acetic acid geraniol ester, 4-hydroxy-4-methyl-2-pentanone, decane, and α-longipinene. However, one volatile, 4-hydroxy-4-methyl-2-pentanone, was released in greater quantities from MEAM1-damaged cabbage plants than from undamaged plants.

## Discussion

We have demonstrated that *B. tabaci* MED prefers undamaged cabbage plants to those damaged by conspecifics. Although the settling and ovipositon behaviours of *B. tabaci* MEAM1 did not significantly differ between MEAM1-damaged and undamaged cabbage plants, MEAM1 whiteflies tended to prefer MEAM1-damaged cabbage plants to undamaged cabbage plants. Furthermore, MEAM1 whiteflies significantly perferred MED-damaged cabbage plants to undamaged cabbage plants. These results may help explain the previous finding that cabbage is a preferred host for MEAM1 but not for MED; once infested by MED but not MEAM1 whiteflies, a cabbage plant will repel many conspecifics.

According to optimal oviposition theory, herbivores preferentially oviposit on plants that provide optimal conditions for offspring development^17^,^18^. Jiao *et al.*^14^ found that female fecundity, female longevity, and nymph survival were greater on cabbage for MEAM1 than for MED whiteflies. The present results are therefore consistent with optimal oviposition theory. This theory is also supported by previous results on the preference of *B. tabaci* and its fitness on the pepper. Jiao *et al.*^14^ found that the effects of the pepper on MEAM1 and MED fitness (adult longevity, fecundity, and nymph survivorship) matched those on settling and oviposition.

The behavioural response of herbivores to host plants damaged by conspecifics varies among herbivores and plants. De Moraes *et al.*^19^, for example, found that females of the moth *Heliothis virescens* were repelled by volatiles emitted from tobacco plants (*Nicotiana tabacum*) infested with conspecific larvae. However, the leaf beetle *Oreina cacaliae* was more attracted to the host plants *Petasites paradoxus* and *Adenostyles alliariae* when they were damaged by conspecifics than when they were undamaged^20^. *B. tabaci* MED’s preference for undamaged cabbage plants over MED-damaged cabbage plants in the current study may be explained by significant differences in the volatile compounds released by the two types of cabbage plants, i.e., perhaps MED is attracted to volatiles from undamaged cabbage plants but repelled by volatiles from MED-damaged plants, because natural enemies may be attracted to the volatiles released from herbivore-damaged plants. Li *et al.*^21^ found that root JA and shoot JA-induced Chinese broccoli volatiles have high efficiencies in attracting the parasitoid *Encarsia formosa* Gahan and the predator *Serangium japonicum*, natural enemies of *B. tabaci.* Therefore, MED may have evolved to avoid such damaged plants to reduce its risk of attack from natural enemies. This is evidently not the case, however, for MEAM1. Although there are no significant differences in the behaviour of MEAM1 on damaged and undamaged plants, MEAM1 whiteflies tend to prefer MEAM1-damaged cabbage plants to undamaged cabbage plants. We also found that MEAM1 whiteflies significantly prefer MED-damaged cabbage plants to undamaged cabbage plants. This means that MEAM1 adults may be using the volatiles emitted by the damaged plants as cue for finding new sources of food, but, in doing so, their risk of exposure to natural enemies increases. This preference is likely to represent a trade-off between the detection of a more apparent host and suboptimal nutritional food quality and the risk of predation or parasitation. Paradoxiaclly, a preference for odours from a suboptimal host, risk of predation or parasitation but detectable and damaged hosts might be a behavioural strategy to cope with uncertainties of host-plant location. Carroll *et al.*^22^ found that fall armyworm (*Spodoptera frugiperda*) larvae preferred odours from damaged over undamaged maize seedlings. As noted earlier, MEAM1 and MED are morphologically identical and are substantially similar genetically, but are reproductively isolated^7^,^23^. Given this reproductive isolation, it seems possible that the effects of MEAM1 and MED damage on volatiles emitted by a host plant and their behavioural responses to the same host plant might be different.

Volatiles often play a key role in the location of hosts by herbivores^19^. It follows that the effect of herbivore injury on the behavior of later-arriving conspecifics may result from herbivore-induced changes in the volatiles released by a plant. In the current study, we found that the quantities of nine volatiles released by MED-infested cabbage plants increased significantly. Previous studies have documented that some plant volatiles repel or attract whiteflies. Wang *et al.*^24^ found that *Chaitophorus salinigra* exhibited taxis in response to benzaldehyde. Cao *et al.*^25^ showed that myrcene attracted *B. tabaci* MEAM1. High levels of p-cymene and curcumene were reported to be strongly repellent to *B. tabaci* MED^26^. In the present study, more benzaldehyde and α-longipinene were released from MED-damaged than undamaged cabbage plants. The different behavioural responses of MEAM1 and MED to undamaged and damaged cabbage plants may be attributed to qualitative differences in the volatile compounds emitted by plants. However, the effects on *B. tabaci* behaviour of the nine volatiles detected in this study requires further investigation.

Nonetheless, the levels of all volatiles detected (except for 4-hydroxy-4-methyl-2-pentanone) did not differ significantly between MEAM1-damaged and undamaged cabbage plants. The lack of a significant change in volatiles has also been reported in other studies involving phloem-feeding insects and host plants. For example, Schwartzberg *et al.*^27^ showed that the pea aphid, *Acyrthosiphon pisum*, did not induce a detectable change in plant volatiles released from broad bean (*Vicia faba*). Turlings *et al.*^28^ documented that even a heavy infestation of the aphid *Rhopalosiphum maidis* failed to induce measurable emissions of volatiles in maize. The effect of 4-hydroxy-4-methyl-2-pentanone on *B. tabaci* behaviour requires further investigation.

Our results show that *B. tabaci* MEAM1 and MED differ in their responses to undamaged cabbage plants and cabbage plants damaged by conspecifics or heterospecifics. The results also show that these differences correspond to differences in the quantity of volatiles released by the damaged plants. To our knowledge, these results provide the first evidence of a possible direct effect of herbivore-induced plant volatiles (HIPVs) on the differing preference of *B. tabaci* MEAM1 and MED for cabbage. Further experiments are needed to determine which volatiles have the most adverse (or beneficial) effect on host-plant selection by whiteflies. The volatiles from such studies may be able to be used as repellents or attractant traps for whiteflies in the field.

## Methods

### Insects and plants

The MEAM1 population of *B. tabaci* used in this study was originally collected from cabbage (*Brassica oleracea* cv. Jingfeng1) in 2004 in Beijing, China. The MED population of *B. tabaci* used in this study was originally collected from poinsettia (*Euphorbia pulcherrima* Wild. ex Klotz.) in 2009 in Beijing. Subsequently, both populations have been maintained on tomato (*L. esculentum* var. Zhongza 9) in separate insect-proof screened cages in a greenhouse at 23 ± 2 °C and with natural lighting. The purity of each population was monitored every two generations by determining the DNA sequence of the haplotypes following amplification by mtCOI primers^28^.

Cabbage (*B. oleracea* var. Jingfeng 1) was grown from seed in a greenhouse at 23 ± 2 °C and with natural lighting. Two weeks after seeds were sown, seedlings were transplanted to 5-cm-diameter plastic pots (one seedling per pot). When they had six main leaves, plants of similar size were selected for each experiment.

### MEAM1 and MED preferences for undamaged cabbage plants vs. cabbage plants damaged by conspecifics and heterospecifics

Bioassays were used to assess the preferences of MEAM1 and MED whiteflies for damaged or undamaged cabbage plants. Damaged plants were obtained by attaching three clip-cages per plant with 100 MEAM1 or MED adults per clip-cage. Undamaged cabbage plants were treated in the same manner, but whiteflies were not added to the clip-cages. The bioassays were conducted as described by Omondi *et al.*^29^ and used cabbage plants of approximately the same size. In brief, two cabbage plants were placed in diagonal corners of a 60 × 40 × 80-cm screen cage (one plant per corner, two per cage); one plant was undamaged, and the other had been damaged by MEAM1, for assays with MEAM1, or by MED, for assays with either MED or MEAM1. The clip cages and whiteflies within them were not removed after the plants were placed in the cages. Approximately 300 assayed MEAM1 or MED adult whiteflies were collected between 7:00 and 8:00 hours and were released near the centre-bottom of the cage. This was accomplished by placing the aspirator sampling bottle containing whiteflies in a clear plastic tumbler in the centre of the cage. Whiteflies moved to and flew out of the open top of the sampling bottle. The number of whiteflies on each plant was determined after 48 h. All of the leaves from each plant were then removed, and the whitefly eggs on each leaf were counted with the aid of a stereomicroscope (Leica, M205C). There were nine replicates for each screen cage. The cages were arranged in blocks in a greenhouse with natural lighting and at ambient temperature (23 ± 2 °C).

### Plant volatile collection and analysis

Cabbage plants were undamaged or were damaged by MEAM1 or MED as described in the previous section. For collection of volatiles emitted, the plants were kept in a greenhouse at 23 ± 2 °C and with 50–70% RH and natural lighting. The plants were transferred individually to large desiccators (40 cm i.d., 60 cm high); an inlet at the top of each desiccator was used to supply pressurised air (100 ml/min) that had been filtered by passage through a jar containing distilled water. Volatiles were collected for 8 h (from 8:00 to 16:00) in a glass tube filled with 150 mg of Tenax resin that had been placed in the outlet of each desiccator. Three plants (one from each treatment) were sampled simultaneously, and each treatment was represented by five replicate plants. The sampling tubes were stored at −20 °C until analysis.

Volatiles were eluted off the Tenax with 2 ml of pentane: diethyl ether (4: 1) and were subjected to gas chromatography-mass spectrometry (GC-MS-2010plus) using a VF-5MS column (0.25 mm × 30 mm, J and W Scientific, Folsom, CA). The temperature programme was as follows: from 40 °C to 95 °C at 3 °C/min, then to 165 °C at 2 °C/min, and finally to 250 °C at 15 °C/min. The volatiles were detected by the MS operated at 70 eV in EI mode. Mass spectra were acquired in full-scan mode (33–400 AMU, 0.4 scan/sec). Compounds were identified by their mass spectra using NIST 2005 (National Institute of Standards and Technology, USA, http:www.nist.gov) and Wiley 9th edition spectral libraries. Relative quantification was based on the peak area of each component of the volatiles.

### Data analysis

The independent samples *t*-test was used to compare whitefly settling and oviposition preference and the relative abundance of individual volatile compounds emitted from undamaged and from MEAM1- or MED-damaged cabbage plants. Statistical analyses were performed with SPSS (version 13.0; SPSS Inc., Chicago, IL, USA).

## Additional Information

**How to cite this article**: Kong, H. *et al.* Differing Behavioural Responses of *Bemisia tabaci* MEAM1 and MED to Cabbage Damaged by Conspecifics and Heterospecifics. *Sci. Rep.* **6**, 35095; doi: 10.1038/srep35095 (2016).

## Figures and Tables

**Table 1:** Peak areas of volatiles released from *B. tabaci* MEAM1-damaged, *B. tabaci* MED-damaged, and undamaged cabbage plants.

Peak areas (×1000)				
Compounds	Retention time	Undamaged plants	MEAM1-damaged plants	MED-damaged plants
alcohols				
2-ethyl-1-hexanol	12.29	41.70 ± 11.28a	49.46 ± 8.47a	181.04 ± 31.56b
benzenemethanol	13.79	10.36 ± 1.52a	8.52 ± 1.75a	41.11 ± 9.44b
aldehydes				
(E)-2-decenol	6.54	6.38 ± 1.80a	11.27 ± 3.22a	44.69 ± 12.67b
benzaldehyde	9.08	58.03 ± 13.94a	111.31 ± 25.49a	193.67 ± 32.45b
nonanal	13.94	38.44 ± 9.94a	30.71 ± 7.65a	152.27 ± 37.87b
esters				
acetic acid geraniol ester	11.37	94.69 ± 18.00a	80.73 ± 16.66a	448.25 ± 131.32b
ketones				
4-hydroxy-4-methyl-2-pentanone	8.87	50.02 ± 13.56a	96.60 ± 13.84b	165.87 ± 24.79b
terpenoids				
α-longipinene	13.59	556.96 ± 131.49a	1008.27 ± 143.93a	2360.18 ± 402.57b
others				
decane	10.93	17.48 ± 3.93a	17.23 ± 4.21a	71.69 ± 11.76b

Values are means ± SE. Means within a row followed by different letters are significantly different at *P* < 0.05.
